# The Emergency nurse Protocols Initiating Care—Sydney Triage to Admission Risk Tool (EPIC-START) trial: protocol for a stepped wedge implementation trial

**DOI:** 10.1186/s43058-023-00452-0

**Published:** 2023-06-20

**Authors:** Kate Curtis, Michael M. Dinh, Amith Shetty, Sarah Kourouche, Margaret Fry, Julie Considine, Ling Li, Thomas Lung, Timothy Shaw, Mary K. Lam, Margaret Murphy, Hatem Alkhouri, Christina Aggar, Saartje Berendsen Russell, Radhika V. Seimon, James A. Hughes, Wayne Varndell, Ramon Z. Shaban

**Affiliations:** 1 Faculty of Medicine and Health, Susan Wakil School of Nursing and Midwifery, The University of Sydney, Camperdown, NSW Australia; 2 Emergency Services, Illawarra Shoalhaven Local Health District, Wollongong Hospital, Crown St, Wollongong, NSW Australia; 3 New South Wales Institute of Trauma and Injury Management, Chatswood, Australia; 4 Sydney Medical School, The University of Sydney, Camperdown, Australia; 5 RPA Green Light Institute for Emergency Care, Sydney Local Health District, Camperdown, Australia; 6 System Sustainability and Performance, NSW Ministry of Health, St Leonards, Australia; 7 Faculty of Medicine and Health, University of Sydney, Camperdown, NSW Australia; 8 Sydney Faculty of Health, University of Technology, Ultimo, NSW Australia; 9 Northern Sydney Local Health District, St Leonards, NSW Australia; 10 School of Nursing and Midwifery and Centre for Quality and Patient Safety Experience in the Institute for Health Transformation, Deakin University, Geelong, VIC Australia; 11 Centre for Quality and Patient Safety Research – Eastern Health Partnership, Box Hill, VIC, Australia; 12 Macquarie University, Sydney, Australia; 13 School of Health and Biomedical Sciences, RMIT University, Melbourne, VIC Australia; 14 Western Sydney Local Health District, North Parramatta, NSW 2145 Australia; 15 Agency for Clinical Innovation, NSW Emergency Care Institute, St Leonards, NSW Australia; 16 Faculty of Medicine, University of New South Wales, Sydney, NSW Australia; 17 Nothern NSW Local Health District, Southern Cross University, Lismore, Australia; 18 School of Nursing, Centre for Healthcare Transformation, Brisbane, QUT Australia; 19 Prince of Wales Hospital Emergency Department, Randwick, Australia; 20 Faculty of Health, University of Technology Sydney, Ultimo, Australia; 21 College of Emergency Nursing Australasia (CENA), Hobart, Australia; 22 Faculty of Medicine and Health, Sydney Institute for Infectious Diseases, The University of Sydney, Camperdown, NSW 2006 Australia; 23 Communicable Diseases Branch, Public Health Unit, Centre for Population Health, Western Sydney Local Health District, North Parramatta, NSW 2141 Australia; 24 New South Wales Biocontainment Centre, Western Sydney Local Health District and New South Wales Health, Westmead, NSW 2145 Australia

## Abstract

**Introduction:**

Emergency department (ED) overcrowding is a global problem and a threat to the quality and safety of emergency care. Providing timely and safe emergency care therein is challenging. To address this in New South Wales (NSW), Australia, the Emergency nurse Protocol Initiating Care—Sydney Triage to Admission Risk Tool (EPIC-START) was developed. EPIC-START is a model of care incorporating EPIC protocols, the START patient admission prediction tool, and a clinical deterioration tool to support ED flow, timely care, and patient safety. The aim of this study is to evaluate the impact of EPIC-START implementation across 30 EDs on patient, implementation, and health service outcomes.

**Methods and analysis:**

This study protocol adopts an *effectiveness-implementation hybrid design* (Med Care 50: 217-226, 2012) and uses a stepped–wedge cluster randomised control trial of EPIC-START, including uptake and sustainability, within 30 EDs across four NSW local health districts spanning rural, regional, and metropolitan settings. Each cluster will be randomised independently of the research team to 1 of 4 dates until all EDs have been exposed to the intervention. Quantitative and qualitative evaluations will be conducted on data from medical records and routinely collected data, and patient, nursing, and medical staff pre- and post-surveys.

**Ethics and dissemination:**

Ethical approval for the research was received from the Sydney Local Health District Research Ethics Committee (Reference Number 2022/ETH01940) on 14 December 2022.

**Trial registration:**

Australian and New Zealand Clinical trial, ACTRN12622001480774p. Registered on 27 October 2022.

**Supplementary Information:**

The online version contains supplementary material available at 10.1186/s43058-023-00452-0.

Contributions to the literature

- Implementation of interventions in emergency department settings is notoriously difficult due to workload unpredictability, high turnover, and undifferentiated nature of emergency department patients.
- Strategies to achieve successful clinician behaviour change and intervention implementation in this complex health care delivery setting are poorly understood.
- This large-scale implementation study will implement and evaluate an emergency model of care in rural, remote, and metropolitan emergency departments in NSW, Australia.
- The acceptability, adoption, appropriateness, costs, feasibility, fidelity, penetration, and sustainability will be tested and refined to inform future strategies for sustained implementation targeting clinician behaviour change in the emergency care context.

## Background

Emergency department (ED) overcrowding is a global problem associated with increasing demand for emergency care services and access block for patients requiring hospital admission [1, 2]. Patients seeking emergency care are routinely confronted by overcrowded waiting rooms and ambulance ramping where the demand for emergency care of clinicians systematically exceeds the supply. An ageing population, increase in chronic condition morbidity, and lack of access to primary care services further exacerbate increased demand for emergency care and ED overcrowding [3, 4].

ED overcrowding leads to treatment delays, reduced adherence to best practice guidelines, increased risk of error, and clinician burnout [3, 4]. Implications for patients are prolonged wait times leading to reduced quality of care and patient dissatisfaction with care. Furthermore, ED overcrowding can exacerbate existing health inequities, with vulnerable populations disproportionately disadvantaged due to increased wait times and reduced access to care.

Emergency clinicians treat patients of all ages, with varying degrees of clinical urgency and severity. Most patients attending the ED have undiagnosed, undifferentiated, and often painful conditions [5]. Delays in the comprehensive assessment, treatment, and escalation of care can be fatal. In Australia, only 63% of patients requiring ‘urgent care’ (i.e. triage category 3) were seen by medical officers within the required 30 min of ED arrival (2020–2021) [2, 6], with emergency nurses solely responsible for the care of these patients during this time. System interventions that aim to address patient flow are important in reducing overcrowding; however, strategies to address the impacts of ED overcrowding must be multi-factorial to ensure patients have access to timely and quality care.

**Fig. 1:**
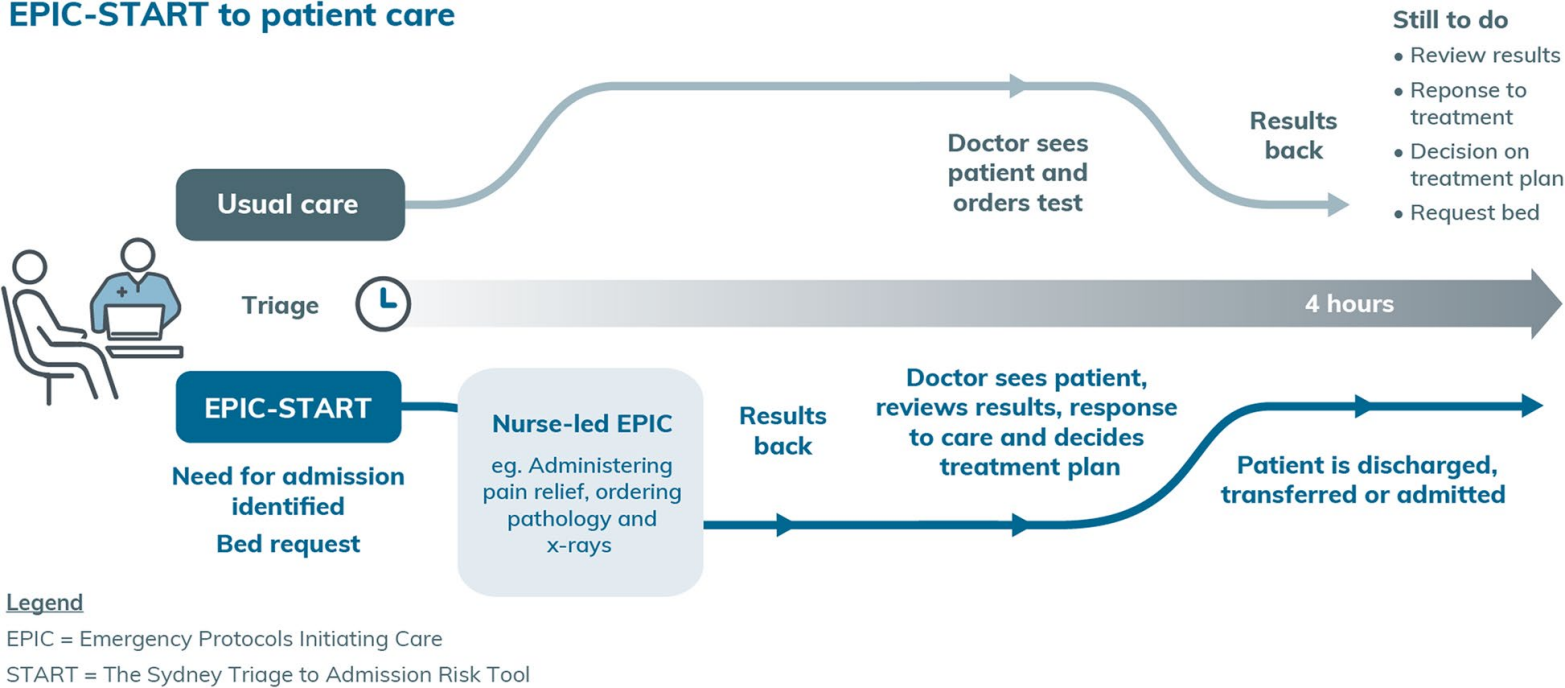
Patient flow with and without EPIC-START

The Emergency nurse Protocol Initiating Care—Sydney Triage to Admission Risk Tool (EPIC-START) is a model of care to optimise ED flow for quality and safety in emergency care (Fig. 1). EPIC-START is founded on systematic reviews of the literature [7, 8], research outcomes [9–11], and deep understanding of the real-world experience of clinical practice, education, and research in emergency care.

The *aim of this study* is to evaluate the EPIC-START system implications across 30 sites in NSW, specifically (i) to evaluate the implementation outcomes: acceptability, adoption, appropriateness, costs, feasibility, fidelity, penetration, and sustainability; (ii) to evaluate the patient and health service outcomes. We hypothesise the use of the EPIC-START model of care in the ED will be associated with (i) reduced patient length of stay, (ii) improved patient time to treatment safety outcomes and clinician experience, and (iii) time to treatment compared to standard care.

## Methods

An effectiveness-implementation hybrid design [12] incorporating a stepped–wedge cluster randomised control trial (SWRCT), including uptake and sustainability, will be conducted to evaluate the EPIC-START model of care. The study is a partnership between academic researchers, clinicians at the sites, and policymakers within the NSW state government and non-government organisations. Reporting has been formatted with Consolidated Standards of Reporting Trials (CONSORT) extension for the SWRCT and SPIRIT (Standard Protocol Items: Recommendations for Interventional Trials) statement [13] (Supplementary materials 1 and 2).

### The intervention

**Fig. 2:**
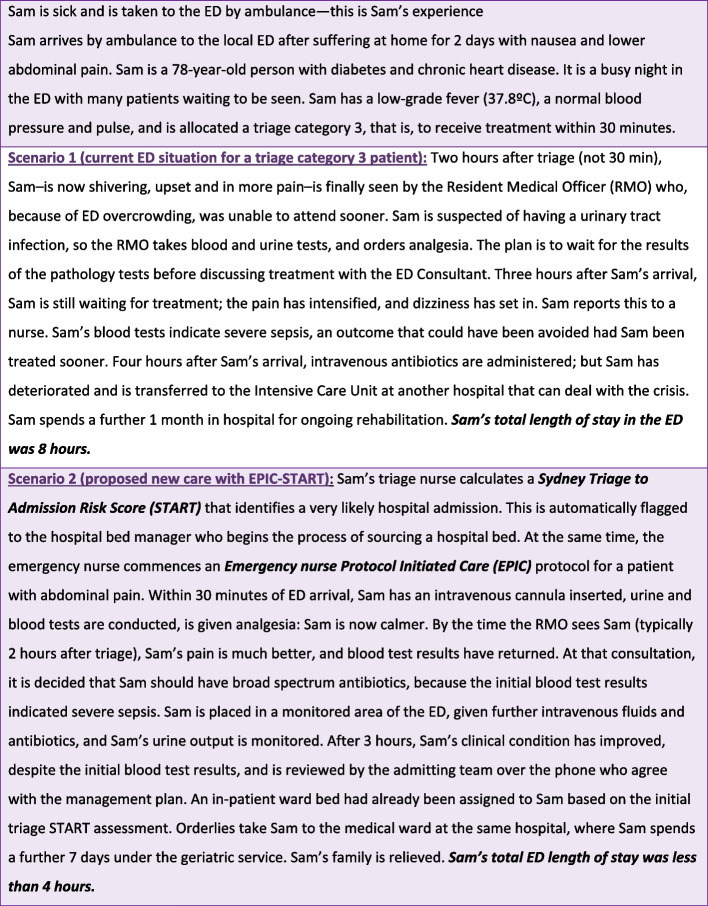
Example of the EPIC-START model of care in action

EPIC-START combines validated data analytic tools with early nurse-initiated care protocols. The EPIC-START model of care incorporates three components: (i) EPIC, (ii) START, and (iii) a deterioration tool. An example of the clinical application of EPIC-START is presented in Fig. 2.

Emergency nurse Protocols Initiating Care (EPIC) is a clinical framework to support the early delivery of standardised evidence-based treatment by emergency nurses for the most common ED presentations. There are 73 EPIC protocols—41 adult and 32 paediatric—which were developed in 2021–2022 by the NSW Agency for Clinical Innovation (ACI) with clinician and policy stakeholders (see NSW Health website). All EPIC protocols are structured on the validated HIRAID™ emergency nursing framework (History, Identify Red flags, Assessment, Interventions, Diagnostics, communication and reassessment) [10]. The EPIC component of EPIC-START is planned for staged state-wide implementation in 2023.

The second component is the Sydney Triage to Admission Risk Tool (START), which is a validated decision support/risk assessment tool at the point of triage [14]. This tool combines the presenting health concern with various other data elements available at the point of triage to estimate the risk of hospital admission or discharge to support senior clinical decisions about streaming and disposition from ED. The START component has been piloted and evaluated in Sydney, NSW [9, 14].

The third element is a clinical deterioration tool that uses an alert system to support earlier detection of, and senior medical response to, patients at risk of clinical deterioration. The clinical deterioration alert system is based on the Modified Early Warning Score (MEWS) [15]. This validated tool estimates the risk of clinical deterioration resulting in death or intensive care unit admission based on vital sign observations routinely recorded by clinicians in the electronic medical record (eMR) observation charts with a reported AUROC of 0.83 (95% CI, 0.83–0.84) [16]. MEWS is one of many early warning scores available that was chosen because of its high sensitivity to the general ED population, and because it uses data that is routinely collected in Australian EDs. Other tools have items such as hypercapnic respiratory failure, which are not routinely collected [17].

### Trial design

The *effectiveness-implementation hybrid design* [12] comprises a closed cohort stepped–wedge cluster randomised controlled trial supported with implementation frameworks to test the implementation strategy and the outcomes of the intervention. A stepped–wedge cluster randomised controlled trial design is considered appropriate for implementation evaluations of health service interventions as it simplifies data collection procedures, supports logistical processes better, aligns with ethical principles, accommodates temporal issues, and optimises financial constraints. The stepped–wedge cluster randomised controlled trial addresses the ethical dilemmas of randomised trial design where essential investigations and/or best-practice treatments may be withheld from control participants [12].

The closed cohort stepped–wedge, cluster randomised controlled trial design involves a sequential rollover of the intervention at four local health district clusters after baseline. This study will be conducted in four local health districts in NSW, Australia. Each cluster will begin in the control condition (baseline data collection) and will sequentially receive the intervention at 6-week intervals.

### Randomisation

Each cluster will be randomised independently by the research team to 1 of 4 dates until all EDs have been exposed. The clusters will be randomised using a computer random number generator to the dates that will implement. Once the initial randomisation of clusters has occurred, all investigators will know the order in which implementation will occur. However, study clusters will be blinded for the analysis.

### Setting

This study will include all patients presenting to one of 30 emergency sites across four local health districts in NSW, Australia (24 EDs/6 multi-purpose services). Multipurpose services provide flexible services to regional and remote communities with integrated health and aged care services [18]. The sites vary in size and are in rural, regional, and urban contexts. The number of sites varies with the geography of each cluster, representing real-world conditions. The sites combined employ approximately 1300 emergency nurses and see over 650, 000 presentations per year (Table 1).

**Table 1:** Local health district (LHD) clusters: ED patient and nurse numbers; patient treatment times; and experience

Cluster	ED patients per year (2020–2021)	Patients (%) whose treatment started within recommended timeframes by triage category			ED nurses	Patient rating (%) ‘very good’ care (2019–2020)
		Cat 2 (10 min), target 80%	Cat 3 (30 min), target 70%	Cat 4 (60 min), target 70%		
Northern NSW (8 EDs, 3 MPS)	198,380	69	70	76	430	70
Sydney LHD (3 EDs)	163,839	48	67	81	402	65
Western Sydney LHD (4 EDs)	197,279	28	52	69	280	48
Southern NSW (8 EDs, 4 MPS)	107,073	66	71	79	180	69
Total (30 EDs/MPS)	666,571	53	65	76	1292	63

*MPS* Multipurpose service, *ED* Emergency department

### Participants

There will be four participant groups in this trial:

1. *ED patients (medical record audits)*: Routinely collected patient-level medical record data including patient presentation information and costing data will be analysed. All patients (adult and paediatric) presenting to the ED during the collection period for each cluster will be included. Patients requiring immediate resuscitation or direct transfers/admissions from other sites will be excluded from the data.
2. *ED nursing staff (surveys)*: All registered nurses permanently employed in the EDs/multi-purpose site across each local health district will be invited to participate in pre- and post-implementation surveys.
3. *ED medical staff (surveys)*: All medical staff employed regularly in the EDs/multi-purpose site across each local health district will be invited to participate in pre- and post-implementation surveys.
4. *ED patients /carers (surveys)*: Any patient with an ED visit during the survey data collection time will be included. A carer may also complete the survey for paediatric patients under 18 years or patients with cognitive disabilities. Patients with a triage category of one (immediate) or presenting for end-of-life care will be excluded, as well as patients or their carers with cognitive impairment, such as intoxication or dementia.

### Study plan

**Fig. 3:**
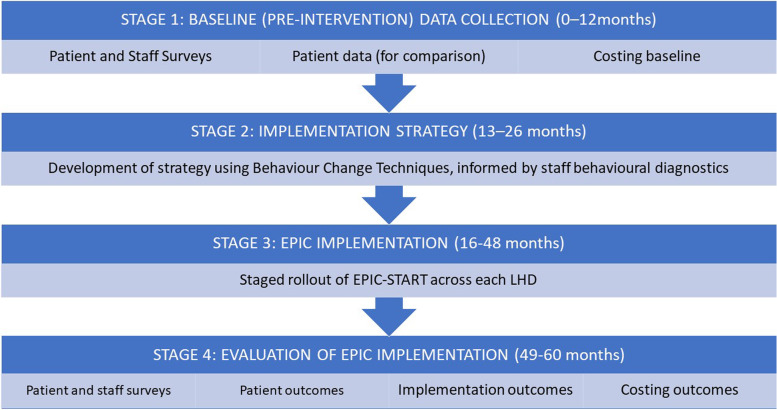
The four stages of the EPIC-START research project

The study will be conducted over four stages (Fig. 3). A logic model depicting the implementation plan has been developed and will be updated after each stage [19].

#### Stage 1: Identify the barriers and facilitators to EPIC-START implementation and use

Behaviour mapping will be undertaken to obtain baseline data prior to the design of a context-specific intervention strategy for EPIC-START. Behaviour mapping will help (1) determine who needs to do what differently, (2) understand the barriers and facilitators for implementation (using the behaviour change wheel [20]), (3) identify the components (behaviours, mode(s) of delivery) that could be overcome to modify the barriers and enhance the facilitators, and (4) identify which interventions can be measured and understood [12]. The Theoretical Domains Framework [21] in conjunction with the Consolidated Framework for Implementation Research (CFIR) [22] will be used to understand the site and individual determinants, i.e. barriers and facilitators to implementation. The CFIR will be used to assess site contexts.

##### Data source

Nursing staff, medical staff, and patient/carer experience pre-implementation surveys will inform stage 1. All data will be managed on a secure platform (REDcap™—Research Electronic Data Capture—https://catalyst.harvard.edu/services/redcap/).

##### Outcomes

The outcomes of stage 1 will be the barriers and facilitators to EPIC-START implementation that are necessary to develop an effective and sustainable EPIC-START implementation strategy (stage 2) that maximises consistent and appropriate uptake. These data will guide the implementation strategy and adaptations that need to be made for implementation. It will also be used to compare sites during evaluation (stage 4) [23].

#### Stage 2: Develop site-specific EPIC-START implementation strategy

An implementation strategy for each cluster will be co-created with site end-users, nurse educators, and the hospital executive through an iterative process guided by the behavioural change wheel (BCW) [20]. The identified aspects of behaviour (including organisational barriers) will be mapped to intervention functions and behaviour change techniques. Intervention functions are ‘broad categories by means of which an intervention can change behaviour’ [20]. We used this approach successfully to test interventions in the ED previously (2021) [10, 24, 25]. The implementation toolkit will be refined for each cluster using mechanisms from the behaviour change techniques taxonomy and the APEASE criteria that consider affordability, practicality, effectiveness/cost-effectiveness, acceptability, side-effects/safety, and equity [20]. Readiness assessments and close consultation with stakeholders at sites and NSW Health will also inform this stage.

##### Sources

The results of the survey from stage 1 and implementation science frameworks (BCW, CFIR).

##### Outcomes

A behaviour change–informed implementation strategy for EPIC-START to be implemented and used in stage 3.

#### Stage 3: Implement EPIC-START

Implementation of the EPIC-START model of care will occur sequentially at the clusters to ensure geographically and clinically diverse ED settings using the strategies identified as effective in stage 2. These strategies will be applied consistently to ensure implementation fidelity*.*

Ongoing implementation support strategies will continue during the implementation stage: for example, the staff at each site will continue to receive support and coaching from the implementation team and updates on the implementation efforts. An implementation nurse will manage each cluster funded by the study. Following implementation, EPIC-START will become part of routine mandatory training to ensure sustainability.

##### Education and training of nursing staff

The education programme will be purposefully designed by educational experts, together with frontline emergency nurses, to frame many of the interventions emergency nurses already use, drawing on the best available evidence regarding patient assessment, risk factors for adverse outcomes, and educational pedagogy to develop deep learning of core concepts and high order thinking. The learning resources (pre-reading, participant workbook, e-learning module, and facilitated interactive workshop) use the educational principles of constructive alignment, backwards design, and scaffolded learning [26–30]. Training in HIRAID™ is a prerequisite to EPIC training in the study sites because effective emergency nursing assessment is essential to patient safety and choosing the correct EPIC protocol to initiate for the undifferentiated patient.

##### Environmental changes

Already brokered agreements with the local health districts will ensure the smooth implementation of EPIC-START. Scoping work around incorporating the START and the deterioration tool within eMR will also be undertaken across sites. In addition, hospital bed managers will be engaged in a brief 1-h education session at each site on the use of the START at triage and its implications for patient flow.

#### Stage 4: Evaluate the effectiveness of EPIC-START

**Fig. 4:**
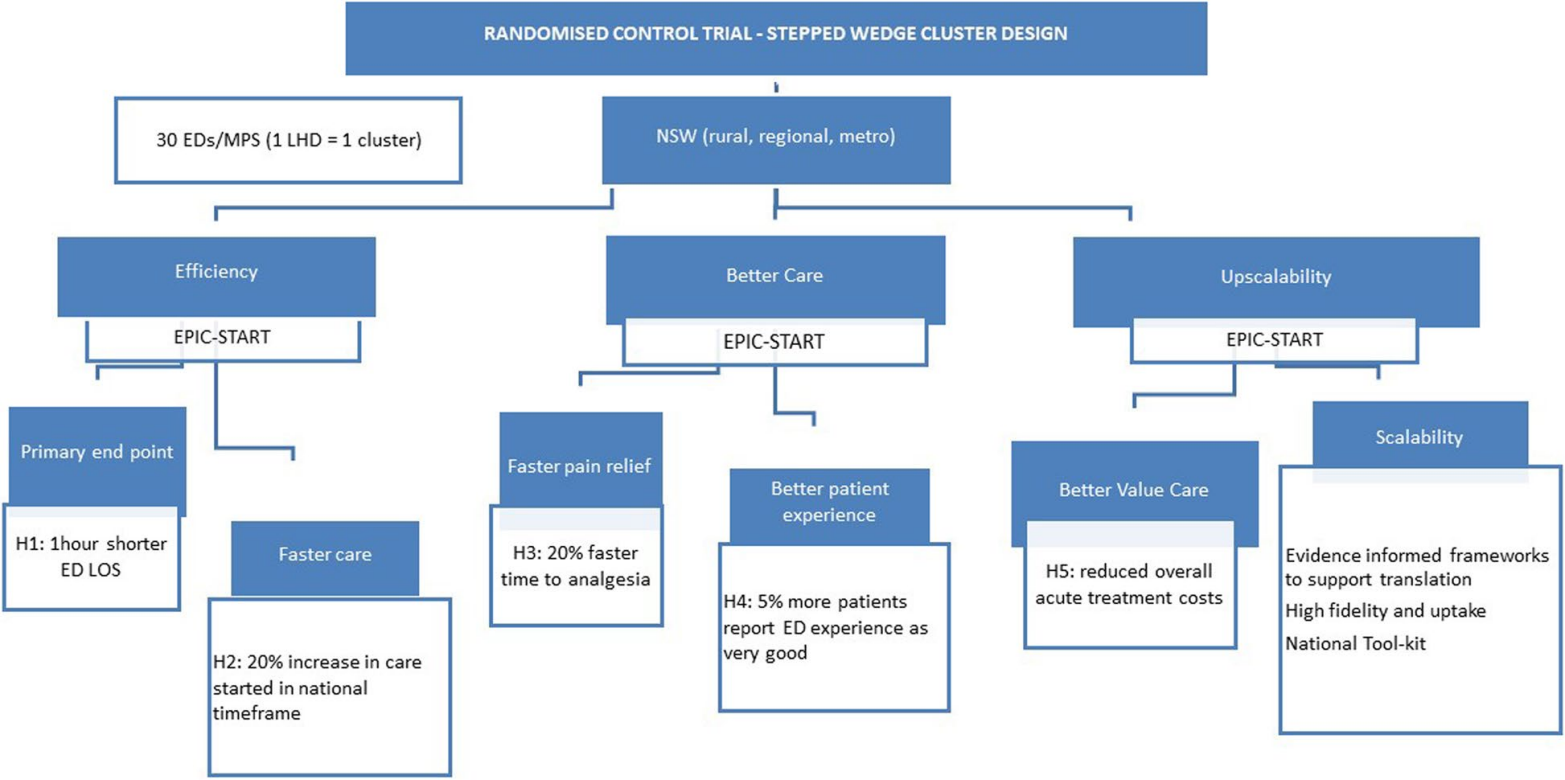
Model of the study with outcomes and hypotheses

The effectiveness of EPIC-START will be evaluated during stage 4 with the question: ‘Will implementing EPIC-START deliver timely and cost-effective care that improves patient health outcomes?’. Five hypotheses will be tested (Fig. 4): As a result of the implementation of EPIC-START:

H1: Patient LOS in ED is 1 h shorter.

H2: There is a 5% increase in care started in the national timeframe.

H3: There is a 20% faster time to analgesia.

H4: 5% more patients report ED experience as ‘very good’.

H5: Overall acute treatment costs are reduced.

##### Sources

Site clinical governance/performance units, electronic medical records, ED performance data, hospital clinical costings, and Incident Information Management System and Performance Unit databases for each of the 30 sites: nursing staff survey, medical staff survey, and patient experience surveys. Implementation logs will be used by the implementation nurses to report on adaptation the implementation log developed using the Framework for Reporting Adaptations and Modifications-Expanded (FRAME) [31].

##### Populations

All participant groups will be used in the evaluation: ED patients, nursing staff, medical staff, and patient experience surveys.

##### Outcomes

Outcomes are summarised in Fig. 4. Outcomes of acceptability, adoption, appropriateness, feasibility, fidelity, implementation cost, penetration, and sustainability will be evaluated [32]. Fidelity is important to control for the differences in intervention delivery between sites and is critical for the internal and external validity of the trial. Implementation fidelity will be measured through a validated tool and informed by site audits and EPIC nurse trainer implementation logs. Descriptive statistics will be used to describe reach, fidelity, and exposure dose to (1) determine acceptability and end-user experience of EPIC-START (i.e. ED clinicians, hospital managers, and consumer representatives) and (2) gain insights on how EPIC-START could be improved and implemented across all Australian EDs.

### Sample size

Sample size calculations considered the estimated between-cluster variance, i.e. between local health district variance, and the design effect associated with the stepped-wedge design [33]. Calculations were based on previous studies and local health district information. Not many previous similar cluster randomised studies have reported the intraclass correlation coefficient (ICC). One similar study on ED LOS used an ICC of 0.18 [34] and another one in community outpatient services but examined waiting time after triage used an ICC of 0.01 [35]. To be conservative, an ICC of 0.05 has been applied in the calculation. Sample sizes relative to the hypotheses are below:

*H1: Patient LOS in ED is 1 h shorter*: The number of ED presentations required per data collection time point at each cluster is 1420 to detect a 1-h change in ED LOS for a two-sided test (80% power; α < 5%), assuming a standard deviation of 5.4 h [36].

H2: There is a 5% increase in care started in the national timeframe: To detect at least a 20% increase for Triage Cat 2 and 3 patients, a total of 1920 patients will be required (120 per local health district per quarter).

H3: There is a 20% faster time to analgesia: To detect at least a 5% reduction in time to analgesia, 960 patients will be required (60 per local health district per quarter). This is achievable for our 20% target.

H4: 5% more patients report ED experience as ‘very good’: To detect at least a 5% increase in the rating as ‘very good’ in the intervention group, compared with the control group, 1920 patients will be required (120 per local health district per quarter).

### Statistical methods

For each outcome of interest, data collected across all measurement periods and all study data collection time points will be used to compare intervention status (pre versus post). Analyses will apply the intention-to-treat principle. Patient data will be analysed according to the status of the study EDs (i.e. pre- or post-intervention) where patients were presented and treated. Outcomes will be assessed at the patient level using mixed effect models, considering the correlation of patient presentation within ED and local health district (cluster) and multiple presentations for the same patient, with adjustment for potential confounding factors. For example, for ED LOS analysis, we will adjust for patient characteristics, e.g. age and gender and clinical characteristic, e.g. number of laboratory tests ordered. The mixed models will also incorporate fixed terms for intervention status and measurement time points (including baseline). The analyses will include multiple time points pre- and post-intervention. The study design will allow us to determine temporal changes in system effectiveness, e.g. if ED LOS continues to decrease over time.

### Cost-effectiveness

The cost-effectiveness analyses will comprise bivariate multi-level models (accounting for relevant confounders including potential secular trends (fixed effects), within site clustering (random effect)) with alpha < 0.05 and correlation between costs and outcomes. Costs related to the EPIC-START (staffing time, education programme) and ED presentations will be incorporated into the analysis using a combination of financial trial records, wage rates, and linked ED data. The incremental cost-effectiveness ratio (ICER) will be calculated from the perspective of the health service to estimate the cost per ‘hour of patient length of stay’ prevented with a time horizon of the hospitalisation period. Discrete event simulation (stochastic) modelling will also be undertaken following best practice recommendations to estimate local health service and Australian system-wide implications (costs and effects) of the sustained implementation of EPIC-START over 5- and 10-year time horizons (discounting at 5%, consistent with Australian recommendations) using trial data and Australian health service profiles to inform assumptions (and probabilistic sensitivity analyses). Economic analyses will be conducted.

## Discussion

This study will undertake a robust and comprehensive examination of the EPIC-START model of care in terms of implementation outcomes (acceptability, adoption, appropriateness, costs, feasibility, fidelity, penetration, and sustainability) and patient and health service outcomes. EPIC-START is designed to enhance nursing and medical practice and system processes, with the aim of more timely patient care, reduced adverse events, and improved patient experience while minimising costs. This trial will deliver ‘real-world’ evidence of the effectiveness and cost-effectiveness of the EPIC-START intervention.

The robust theoretical underpinnings of the design and feasibility and pilot investigations are strengths of this trial that will also enable testing the impact of the intervention and the mechanisms of action of the intervention strategies, contributing empirical evidence to the theoretical foundations of implementation science. The EPIC-START trial will provide important evidence for systems and clinician behaviour change interventions in the ED setting, which is a unique environment for implementation.

**Fig. 5:**
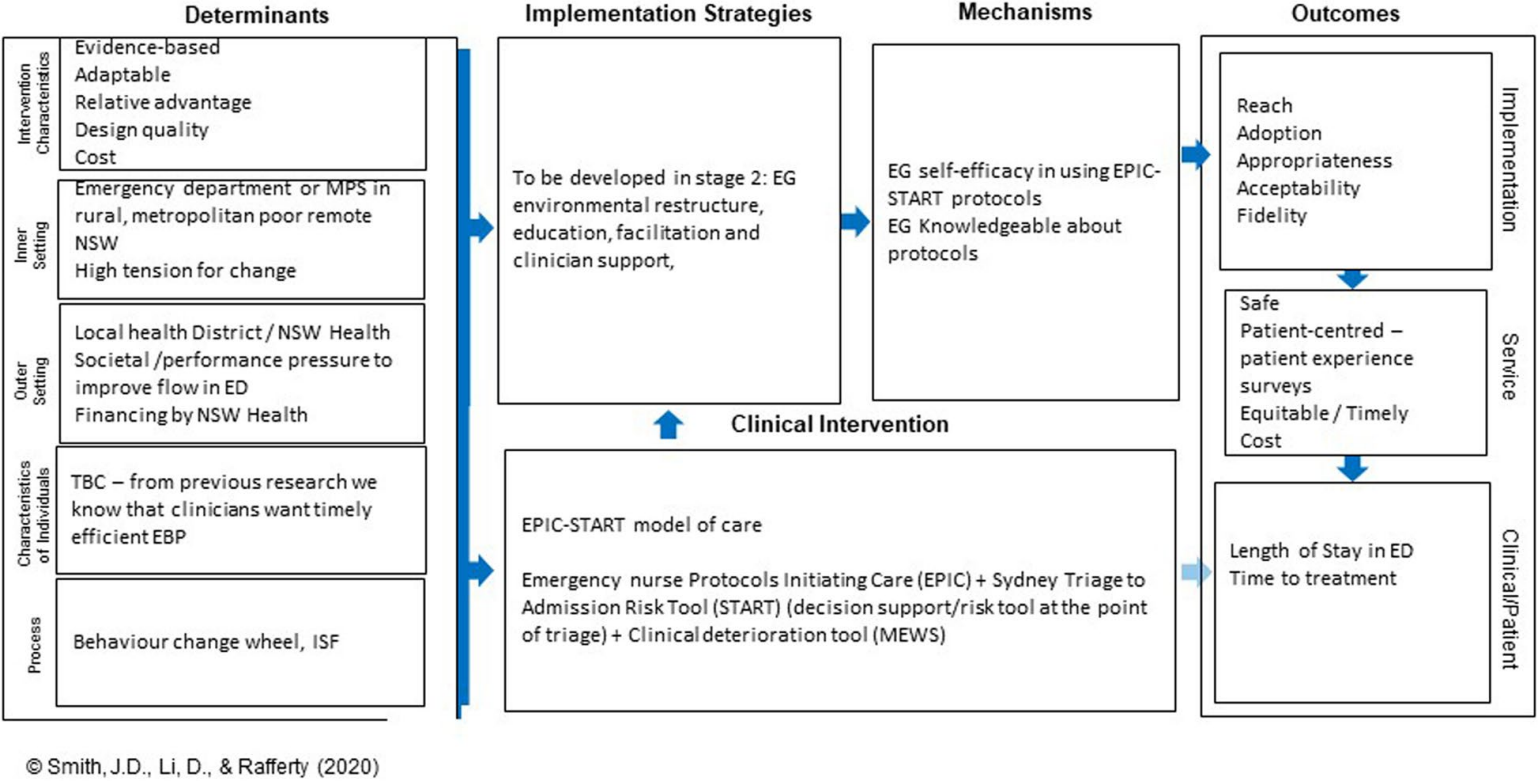
Logic model for the EPIC-START study

This study includes a detailed plan to identify contextual barriers, implement strategically to ameliorate context barriers, and evaluate the implementation. A logic model details this plan (Fig. 5). A process evaluation will assess the implementation outcomes and adaptations, adding further understanding regarding how to implement evidence-based practice in real-world settings.

The implementation evaluation will result in rich clinical, implementation, and economic evidence to accelerate system-wide change and transform emergency clinical practice and care delivery, while adding to the empirical evidence to support further theoretical developments in implementation science.

### Methodological considerations and risks

There are foreseeable risks to the implementation and evaluation of EPIC-START in this study, such as changes to the electronic medical record systems, COVID-19 and natural disasters, and competing projects, for which mitigation strategies have been established. Partner agreements were obtained as part of the study proposal/design and co-design of implementation strategies with clinicians and service leaders to reduce risk. Other risks may be delays in the publication of EPICs, integration and uptake of the SMART, and deterioration tools into eMR and clinician practice.

## Conclusion

Globally, ED patients experience significant treatment and care delays associated with ED overcrowding. Hence, we plan on implementing and evaluating the EPIC-START model of care in EDs in NSW, Australia. Our trial will inform further upscale and spread of the EPIC-START and other evidence-based interventions into EDs nationally and worldwide in future implementation studies.

## Supplementary Information

**Additional file 1: Supplementary file 1. **SW RCT _checklist_completedR0

**Additional file 2: Supplementary file 2. **SPIRIT_checklistR0

**Additional file 3: Supplemantary file 3. **Ethics approvalR0

**Additional file 4: Supplementary file 4. **Grant fundingR0.

## Data Availability

Not applicable.
