# Supplementary material for: The Emergency nurse Protocols Initiating Care—Sydney Triage to Admission Risk Tool (EPIC-START) trial: protocol for a stepped wedge implementation trial
Source: Implement Sci Commun. 2023 Jun 20;4:70. doi: 10.1186/s43058-023-00452-0 (PMC10280960; doi:10.1186/s43058-023-00452-0)
Supplement: Supplementary file 4 — Additional file 4: Supplementary file 4. Grant fundingR0. [file 43058_2023_452_MOESM4_ESM.pdf]

RESEARCH SUPPORT SCHEME SCHEDULE

This Schedule forms part of, and must be read in conjunction with, the terms of the Agreement regarding provision of Funding from the MRFF for Research Activities as executed by the Commonwealth and the Eligible Organisation and administered by NHMRC for Health and as most recently varied (Agreement). Capitalised terms in this Schedule have the same meaning as in the Agreement.

The Commonwealth and the Eligible Organisation agree that this Schedule covers a single Research Activity.

This offer must be accepted by 08 April 2022 or the offer may lapse unless NHMRC, on behalf of Health, notifies the Eligible Organisation in writing otherwise.

A. TYPE OF FUNDING

2022 Models of Care to Improve the Efficiency and Effectiveness of Acute Care Grant Opportunity

The MRFF provides grants of financial assistance to support health and medical research and innovation in improving the health and wellbeing of Australians. It operates as an endowment fund with the capital preserved in perpetuity. The MRFF reached maturity at $20 billion in July 2020. The MRFF provides a long-term sustainable source of funding for endeavours that aim to improve health outcomes, quality of life and health system sustainability.

Funding for this Research Activity is provided under the Emerging Priorities and Consumer Driven Research Initiative. More information on the initiative is available in the Grant Guidelines, available at https://www.grants.gov.au/.

The expected outcomes of the 2022 Models of Care to Improve the Efficiency and Effectiveness of Acute Care Grant Opportunity is to improve the health and wellbeing of Australians by generating evidence that supports implementation of more effective and timely acute care approaches.

In accepting the Funding, the Eligible Organisation agrees to ensure that the Research Activity is conducted in accordance with the terms of this Agreement, including this Schedule.

B. FUNDING PERIOD

The Funding Period for this Research Activity is 5 year(s).

Commencement date for the Research Activity:	01 April 2022
End date for the Research Activity:	31 March 2027

C. RESEARCH ACTIVITY

Funding Identification Number:	MRF2017845. This number identifies the Application submitted for this Research Activity.
Eligible Organisation:	University of Sydney

Title:	Giving patients an EPIC-START: An evidence based, data driven model of care to improve patient care and efficiency in emergency departments
Research Activity objectives and outcomes:	The objectives and outcomes specified in the Application (as approved by Health) for this Research Activity.
Institutional approvals required:	All Eligible Organisation Approvals (as required by all applicable NHMRC Approved Standards and Guidelines) as well as any approvals that the relevant Funding Policy requires the Eligible Organisation or its Personnel to obtain.

D. SPECIFIED PERSONNEL

Chief Investigator A

CIA - Professor Kate Curtis

Chief Investigator(s)

Professor Ramon Shaban

Doctor Amith Shetty

Doctor Hatem Alkhouri

Associate Professor Christina Aggar

Wayne Varndell

Doctor Thomas Lung

Professor Margaret Fry

Doctor James Hughes

Associate Professor Ling Li

Associate Professor Michael Dinh

Doctor Margaret Murphy

Doctor Sarah Kourouche

Professor Julie Considine

Professor Timothy Shaw

E. FUNDING

Total GST-exclusive Funding amount is: **$ 2,847,592.24**.

F. NOT USED

G. OTHER CONTRIBUTIONS

If any Other Contributions are specified in the Application for this Research Activity, the Eligible Organisation is required to ensure that all of those Other Contributions are provided for this Research Activity in the amount and in the manner (including any times) specified in the Application (as approved by Health).

H. FUNDING CONDITIONS

The Eligible Organisation must expend the Funding to perform the Research Activity as described in Item C of this Schedule and in accordance with the Agreement.

Other Funding Conditions for this Research Activity are set out in Attachment 1 to this Schedule.

I. GST-EXCLUSIVE ALLOCATION OF FUNDS OVER FUNDING PERIOD

**Year**	**2021-22**	**2022-23**	**2023-24**	**2024-25**	**2025-26**	**2026-27**
**TOTAL**	$ 323,800.00	$ 400,000.00	$ 649,518.45	$ 904,755.35	$ 569,518.44	$0.00

Note:

- GST may be applied to some components of the above.
- No indexation is payable.
- The first payment will be made within 45 days of schedule execution and annually thereafter.

J. NOTICES

a) NHMRC:
Research Administration Section
National Health and Medical Research Council
GPO Box 1421
Canberra City ACT 2601
mrff.postaward@nhmrc.gov.au

Attachment 1 to Schedule

H.1 Vulnerable Persons

H.1.1 In this Agreement:

**Criminal or Court Record**	means any record of any Other Offence;
**Other Offence**	means, in relation to a person, a conviction, finding of guilt, on-the-spot fine for, or court order relating to:<br>(a) an apprehended violence or protection order made against the person;<br>(b) the consumption, dealing in, possession or handling of alcohol, a prohibited drug, narcotic or other prohibited substance;<br>(c) violence against another person or the injury, but excluding the death, of another person; or<br>(d) an attempt to commit a crime or offence, or to engage in any conduct or activity, described in paragraphs (a) to (c);
**Police Check**	means a formal inquiry made to the relevant police authority in each State or Territory and designed to obtain details of an individual's criminal conviction or a finding of guilt in all places (within and outside Australia) that the Eligible Organisation know the person has resided in;
**Serious Offence**	means:<br>(a) a crime or offence involving the death of a person;<br>(b) a sex-related offence or a crime, including sexual assault (whether against an adult or child); child pornography, or an indecent act involving a child.

	(c) fraud, money laundering, insider dealing or any other financial offence or crime, including those under legislation relating to companies, banking, insurance or other financial services; or<br><br>(d) an attempt to commit a crime or offence described in (a) to (c);
**Serious Record**	means a conviction or any finding of guilt regarding a Serious Offence;
**Vulnerable Person**	means an individual aged 18 years and above who is or may be unable to take care of themselves, or is unable to protect themselves against harm or exploitation for any reason, including age, physical or mental illness, trauma or disability, pregnancy, the influence, or past or existing use, of alcohol, drugs or substances or any other reason.

H.1.2 Before any person commences performing work on any part of the Research Activity that involves working or contact with a Vulnerable Person, the Eligible Organisation must:

(a) obtain a Police Check for that person;

(b) confirm that the person is not prohibited by any law from being engaged in a capacity where they may have contact with a Vulnerable Person;

(c) comply with all State, Territory or Commonwealth laws relating the employment or engagement of persons in any capacity where they may have contact with a Vulnerable Person; and

(d) ensure that the person holds all licences or permits for the capacity in which they are to be engaged, including any specified in the Schedule, and the Eligible Organisation must ensure that Police Checks and any licences or permits obtained in accordance with this Item H.1.2 remain current for the duration of their involvement in the Research Activity.

H.1.3 The Eligible Organisation must ensure that a person does not perform work on any part of the Research Activity that involves working or contact with a Vulnerable Person if a Police Check indicates that the person at any time has:

(a) a Serious Record; or

(b) a Criminal or Court Record and the Eligible Organisation has not conducted a risk assessment and determined that any risk is acceptable.

H.1.4 In undertaking a risk assessment under Item H.1.3, the Eligible Organisation must have regard to:

(a) the nature and circumstances of the offence(s) on the person's Criminal or Court Record and whether the charge or conviction involved Vulnerable Persons;

(b) whether the person's Criminal or Court Record is directly relevant to, or reasonably likely to impair the person's ability to perform, the role that the person will, or is likely to, perform in relation to the Research Activity;

(c) the length of time that has passed since the person's charge or conviction and his or her record since that time;

(d) the circumstances in which the person will, or is likely to, have contact with a Vulnerable Person as part of the Research Activity;

(e) any other relevant matter,

and must ensure it fully documents the conduct and outcome of the risk assessment.

H.1.5 The Eligible Organisation agrees to notify Health of any risk assessment it conducts under this Item H.1 and agrees to provide Health with copies of any relevant documentation on request.

H.1.6 If during the term a person involved in performing work on any part of the Research Activity that involves working or contact with a Vulnerable Person is:

(a) charged with a Serious Offence or Other Offence, the Eligible Organisation must immediately notify Health; or

(b) convicted of a Serious Offence, the Eligible Organisation must immediately notify Health and ensure that that person does not, from the date of the conviction, perform any work or role relating to the Research Activity.

H.2 Not Used

H.3 Reporting

In addition to the standard reporting listed in clause 10 of the Agreement, the Eligible Organisation must provide the additional reports at H.3.1, H.3.2 and H.3.3. The Commonwealth reserves the right to amend or adjust the requirements as required.

H.3.1 Progress reporting

The Eligible Organisation must report on the progress of the Research Activity by submitting a progress report every 12 months from the commencement of the grant. This information will include:

- progress towards completion of agreed research activities, including any risks arising and how these are being managed to ensure project outcomes

- where applicable, evidence to demonstrate progress against the outcome/s and result/s identified in the Measures of Success statement
- total expenditure incurred to date, including evidence
- information that supports evaluation of the MRFF.

Grantees are required to use the reporting template available on the NHMRC website to complete these reports.

H.3.2 Final Report

Within six months of the end of the Research Activity, the Eligible Organisation must provide an end of project report that includes:

- evidence of completion of agreed research activities as specified in the Agreement (including, but not limited to, evidence of impact)
- where applicable, evidence to support achievement of the outcome/s and result/s identified in the Measures of Success statement
- total expenditure incurred
- a declaration that the grant money was spent in accordance with the Agreement and a report on any underspends of the grant money
- information that supports evaluation of the MRFF.

Grantees are required to use the reporting template available on the NHMRC website to complete these reports.

H.3.3 Ad-hoc reporting

The Commonwealth may ask the Grantee for ad-hoc reports on the project. The Grantee must provide these reports in the timeframes notified by the Commonwealth. The Grantee may also be asked to participate in and provide information about the Activity as part of the overarching evaluation of the MRFF.

H. 4 Not used

H.5 Not used

H.6 Interest

H.6.1 The Funding for this Research Activity includes any interest that the Eligible Organisation (or a Participating Institution) earns on the Funding and the Eligible Organisation (and Participating Institution) must account for that interest as part of the Funding.

H.7 Other

H.7.1 The Eligible Organisation must, within 12 months of completion of the Research Activity, disseminate the research findings and de-identified research data arising out of the Research Activity, in accordance with best practice and through:

(a) ensuring that research findings are available in an open access repository in accordance with clause 13.9 and 13.10 of the Agreement;

(b) content specific forums;

(c) submitting to peer-reviewed journals; and

(d) making available as lay summaries concurrently with sharing and dissemination of research results.

H.7.2 Funding provided under this Agreement for this Research Activity cannot be used to fund activities for which funding has been obtained from another government source (including another part of the Commonwealth) for the same activities or purpose.
