# Supplementary material for: The Emergency nurse Protocols Initiating Care—Sydney Triage to Admission Risk Tool (EPIC-START) trial: protocol for a stepped wedge implementation trial
Source: Implement Sci Commun. 2023 Jun 20;4:70. doi: 10.1186/s43058-023-00452-0 (PMC10280960; doi:10.1186/s43058-023-00452-0)
Supplement: Supplementary file 3 — Additional file 3: Supplemantary file 3. Ethics approvalR0 [file 43058_2023_452_MOESM3_ESM.pdf]

ADDRESS FOR ALL CORRESPONDENCE
RESEARCH ETHICS AND GOVERNANCE OFFICE
ROYAL PRINCE ALFRED HOSPITAL

TELEPHONE: (02) 9515 6766
EMAIL: SLHD-RPAEthics@health.nsw.gov.au
REFERENCE: X22-0354 & 2022/ETH01940

14 December 2022

This letter constitutes ethical approval only. You must NOT commence this research project at ANY site until you have submitted a Site Specific Assessment Form to the Research Governance Officer and received separate authorisation from the Chief Executive or delegate of that site.

Dear Professor Curtis,

Re: Protocol no. X22-0354 & 2022/ETH01940 - "Implementation evaluation of the Emergency nurse Protocol Initiating Care - Sydney Triage to Admission Risk Tool (EPIC-START) model of care"

The Executive of the Ethics Review Committee, at its meeting of 14 December 2022 considered your correspondence of 2 and 9 December 2022. In accordance with the decision made by the Ethics Review Committee, at its meeting of 9 November 2022, ethical approval is granted.

I am pleased to advise that final ethical approval has been granted on the basis of the following:

- The research project meets the requirements of the *National Statement on Ethical Conduct in Human Research*.

- The Committee granted a waiver of the usual requirement for the consent of the individual for the use of their health information in a research project, in accordance with the Health Records and Information Privacy Act 2002 (NSW) and the NSW Privacy Commissioner's Statutory guidelines on research and the NHMRC Guidelines approved under Section 95A of the Privacy Act 1988.

This approval includes the following:

- HREA (Version 3, 8 December 2022)
- Protocol (Version 3, 9 December 2022)
- List of EPIC Protocols (undated)
- Participant Information Sheet – Clinician (Version 2, 5 December 2022)
- Participant Information Sheet – Patient (Version 2, 5 December 2022)
- Interview Script – Patient/Carer (Version 1, 25 October 2022)
- Nursing Survey (Version 2, 05 December 2022)
- Medical Staff Survey (Version 2, 05 December 2022)

- Patient Survey (Version 1, 25 October 2022)
- Data Variables (Version 1, 25 October 2022)
- Implementation Log (Version 1, 25 October 2022)
- Recruitment Advertisement (Version 1, 25 October 2022)
- Implementation Fidelity Assessment (Version 1, 25 October 2022)
- SLHD Privacy Compliance Form (Version 1, 25 October 2022)
- USYD Privacy and Confidentiality Clause (Version 1, 25 October 2022)
- Research Data Management Plan (Version 1, 18 October 2022)
- USYD Research Data Management Plan (Dated 11 August 2022)
- Start Tool (Version 1, 25 October 2022)
- Letters of Support (dated 23 February 2022)
- ACI Protocol – Abdominal Pain (undated)
- GCP Certificate – Amith Shetty (dated 29 November 2022)
- GCP Certificate – Christina Aggar (dated 18 November 2022)
- GCP Certificate – Hatem Alkhouri (dated 18 November 2022)
- GCP Certificate – Julie Considine (dated 29 September 2021)
- GCP Certificate – Kate Courtis (dated 20 August 2021)
- GCP Certificate – Kylie Smith (dated 27 November 2022)
- GCP Certificate – Ling Li (dated 18 November 2022)
- GCP Certificate – Louise Case (dated 17 November 2022)
- GCP Certificate – Margaret Fry (dated 19 November 2022)
- GCP Certificate – Margaret Murphy (dated 29 November 2022)
- GCP Certificate – Mary Lam (dated 17 November 2022)
- GCP Certificate – Michael Dinh (dated 14 August 2021)
- GCP Certificate – Radhika Seimon (dated 11 November 2021)
- GCP Certificate – Ramon Zenel Shaban (dated 18 November 2022)
- GCP Certificate – Saartje Berendsen Russell (dated 07 June 2021)
- GCP Certificate – Sarah Kourouche (dated 15 November 2022)

- GCP Certificate – Tom Lung (dated 21 November 2022)

- GCP Certificate – Tim Shaw (dated 18 November 2022)

You are asked to note the following:

The Committee noted that authorisation will be sought to conduct the study at the following sites:

- Royal Prince Alfred Hospital
- Concord Hospital
- Canterbury Hospital
- Balmain Hospital
- Auburn Hospital
- Blacktown Hospital
- Mount Druitt Hospital
- Westmead Hospital
- Crookwell Hospital
- Goulburn Hospital
- Yass Health Service
- Queanbeyan Health Service
- Braidwood (MPS)
- Batemans Bay Hospital
- Moruya Hospital
- Cooma Health Service
- Bega South-East Regional Hospital
- Bombala Hospital (MPS)
- Ballina District Hospital
- Bonalbo MPS
- Byron Central Hospital
- Casino & District Memorial Hospital
- Grafton Base Hospital
- Kyogle (MPS)
- Lismore Base Hospital
- Maclean District Hospital
- Murwillumbah District Hospital
- Nimbin (MPS)
- The Tweed Hospital
- Urbenville (MPS)

- It is a requirement of ethics approval that, before its commencement, this clinical trial is registered on a publicly accessible register, such as the Australian New Zealand Clinical Trials Registry or another appropriate international register. The Committee therefore sought details of the Register in which the study has been included and its registration number.

- This approval is valid for **five years**, and the Committee requires that you furnish it with annual reports on the study's progress beginning in **December 2023.** This will be through the submission of a milestone in REGIS.

- This human research ethics committee (HREC) has been accredited by the NSW Department of Health as a lead HREC under the model for single ethical and scientific review and is constituted and operates in accordance with the National Health and Medical Research Council's *National Statement on Ethical Conduct in Human Research* and the *CPMP/ICH Note for Guidance on Good Clinical Practice*.

- In accordance with the National Statement, Chapter 4.7, you are reminded that you must seek ethical approval from the HREC of the Aboriginal Health and Medical Research Council (AHMRC) if you intend to use Aboriginal or Torres Strait Islander status in any presentation or publication.

- **Partnering with Consumers:** As per Standard 2 of The National Clinical Trials Governance Framework, you are asked to provide an annual update with your annual progress report (milestone) on the ongoing involvement of consumers in the planning, design, delivery, measurement and evaluation of the trial.

- **Good Clinical Practice (GCP):** When adding additional sites, it is a condition of approval that the GCP Certificate of Completion be submitted for the principal investigator responsible for the new site.

- You must immediately report anything which might warrant review of ethical approval of the project in the specified format, including unforeseen events that might affect continued ethical acceptability of the project.

- You must notify the HREC of proposed changes to the research protocol or conduct of the research in the specified format.

- You must notify the HREC and other participating sites, giving reasons, if the project is discontinued at a site before the expected date of completion.

Should you have any queries about the Committee's consideration of your project, please contact me. The Committee's Terms of Reference, Standard Operating Procedures, membership and standard forms are available from the Sydney Local Health District website.

<u>If you are not using REGIS, a copy of this letter must be forwarded to all site investigators for submission to the relevant Research Governance Officer.</u>

The Ethics Review Committee wishes you every success in your research.

Regards,

S.Thomas

Sanaa Thomas
Executive Officer
Clinical Trials Sub-committee

For:
Rosemary Carney
Executive Officer
Ethics Review Committee (RPAH Zone)
